# A subnational reproductive, maternal, newborn, child, and adolescent health and development atlas of India

**DOI:** 10.1038/s41597-023-01961-2

**Published:** 2023-02-10

**Authors:** Carla Pezzulo, Natalia Tejedor-Garavito, Ho Man Theophilus Chan, Ilda Dreoni, David Kerr, Samik Ghosh, Amy Bonnie, Maksym Bondarenko, Mihretab Salasibew, Andrew J. Tatem

**Affiliations:** 1 WorldPop, School of Geography and Environmental Science, University of Southampton, Highfield Campus, Southampton, SO17 1BJ UK; 2 School of Mathematical Sciences, University of Southampton, Southampton, SO17 1BJ UK; 3 Social Statistics & Demography, University of Southampton, Highfield Campus, Southampton, SO17 1BJ UK; 4 Children’s Investment Fund Foundation (CIFF), London, UK

**Keywords:** Developing world, Social sciences

## Abstract

Understanding the fine scale and subnational spatial distribution of reproductive, maternal, newborn, child, and adolescent health and development indicators is crucial for targeting and increasing the efficiency of resources for public health and development planning. National governments are committed to improve the lives of their people, lift the population out of poverty and to achieve the Sustainable Development Goals. We created an open access collection of high resolution gridded and district level health and development datasets of India using mainly the 2015–16 National Family Health Survey (NFHS-4) data, and provide estimates at higher granularity than what is available in NFHS-4, to support policies with spatially detailed data. Bayesian methods for the construction of 5 km × 5 km high resolution maps were applied for a set of indicators where the data allowed (36 datasets), while for some other indicators, only district level data were produced. All data were summarised using the India district administrative boundaries. In total, 138 high resolution and district level datasets for 28 indicators were produced and made openly available.

## Background & Summary

Reproductive, maternal, newborn, child health and development, adolescent’s health, climate change, ending poverty and hunger and promoting gender equality and literacy among boys and girls are all central to the Sustainable Development Goals (SDG) agenda for 2030. With the commitment of World leaders who pledged common action and endeavour across such a broad and universal policy agenda, SDGs have the ambition of building a better future for all people, achieving improved health and quality of life of current and future generations, implement sustainable development and equal access to health for all, and leaving no one behind^1^.

In India, women and children comprise approximately 70% of the population^2^. As part of its interventions at national and sub-national levels, the Ministry of Women and Child Development promotes social and economic empowerment of women and the care, development, and protection of children^3^. However, despite the gains over the last three decades, uplifting the condition of women and children remains a challenge^4,5^.

Over recent decades, the Government of India has shown a commitment to addressing several development concerns, especially those affecting children, adolescents, and women^4^. Through a series of initiatives in the context of its national development agenda, the Government has successfully lifted more than 250 million people out of multidimensional poverty through economic growth and empowerment^6,7^, improved health and sanitation conditions, electricity and housing as well as nutrition and education among vulnerable populations and enhanced social inclusion and social protection in the country^8^. Moreover, it is widely recognised that there is an association between air pollution and adverse health outcomes^9,10^, and increasingly studies have investigated the impact of the burden of air pollution on the economy^11^. Climate action strategies for clean and efficient energy systems have been put in place^8,12^, and progress observed towards the achievement of the climate-related SDGs (SDG 13)^1^. However India presents wide variations between and within states in terms of the effects of air pollution on health and the economy^11^.

Despite progress in all areas and while the reforms implemented to achieve the SDGs have reduced the disparities across many socio-economic, health and environmental indicators, within country inequalities are still widespread^13–15^. The country is ranked 120 out of 193 UN Member States, with a score of 60.07, where the score measures a country’s total progress towards achieving all 17 SDGs and a score of 100 signifies that all goals have been achieved^16,17^.

Regional level studies have shown heterogeneities in maternal, newborn and child health indicators, and inequalities in child undernutrition and in access to health care affect the most vulnerable groups in the country^15,18–21^. Some areas of India still lag behind on women’s education, economic empowerment, access to maternal and child health services, child mortality and malnutrition^22^. When looking at individual health and development indicators, data from the NFHS-4 survey show how inequalities persist. For example, there is a difference of 48 percentage points between women in the richest quintile (73%) and those in the poorest quintile (25%) in the percentage of women attended four or more times during pregnancy by any provider^23^.

Reproductive, maternal, newborn, child, and adolescent health and development indicators are essential to track progress towards the SDGs and to inform development policies, ensuring that no one is left behind. Monitoring progress towards the SDGs for 2030 is typically done at national level^24,25^, while concerns about health and wealth inequity indicate that there is a need for analysis of health indicators at the microgeographic level or for population subgroups^15^. With geospatial approaches being used to produce fine scale estimates of SDG-related indicators, sub-national maps are now widely produced to support planning and implementation of health and development interventions in different settings, and geographically disaggregated information are increasingly serving the targeting of resources and more precise policy applications^26–28^.

Here, using the most recent sources of data at the time of writing, including household surveys and other openly available data sources, we assembled a collection of subnational reproductive, maternal, newborn, child, and adolescent health and development indicators for India, to support policy and planning activities and to improve geographic targeting towards the achievement of the SDGs. A health and development atlas consisting of a collection of 138 datasets for 28 indicators at subnational scales, including estimated 5 km × 5 km high-resolution maps of India with relative prediction uncertainties mapped, as well as district level maps of India, was assembled to support the review of development and health strategies and inform future actions.

## Methods

Gridded estimates of selected reproductive, maternal, newborn, child, and adolescent health and development indicators were produced for India at a spatial resolution of 5 km. Where the construction of gridded estimates was not feasible, district level estimates were produced.

**Fig. 1:**
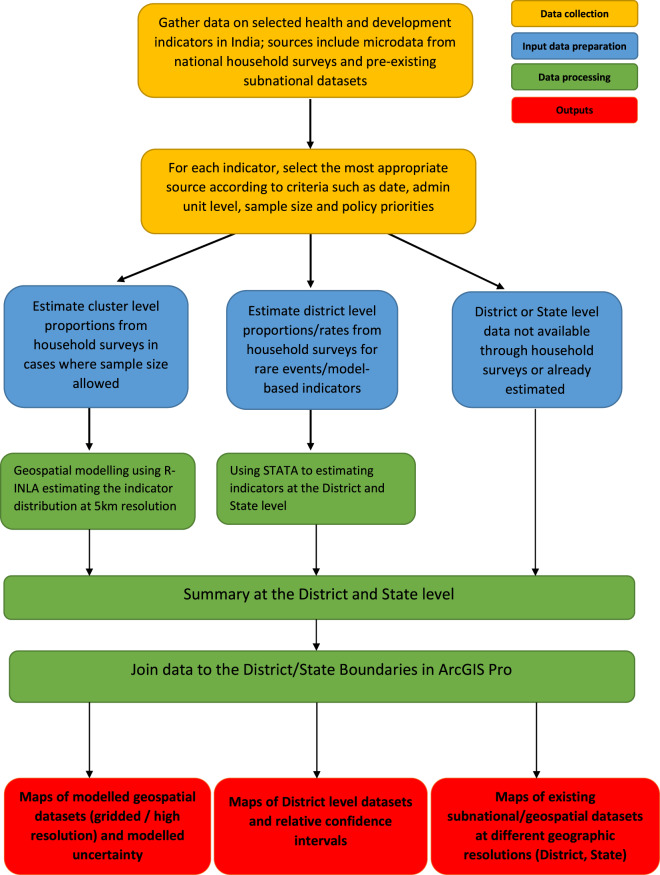
Schematic overview of the data processing method adopted to generate 5 km × 5 km high resolution and district level reproductive, maternal, newborn, child, and adolescent health and development datasets in India.

The indicators mapped in this work were collected from a range of sources, including geolocated and nationally and sub-nationally representative household surveys and pre-existing subnational datasets. These covered indicators on child, adolescent and women’s health, nutrition, and wellbeing, as well as selected climatic indicators. For each indicator, the most appropriate data source was selected, according to criteria such as date, administrative level unit, sample size and policy priority. Where possible, for selected indicators and using the latest available household survey for India at the time of writing, geospatial modelling techniques were applied to estimate 5 km spatial resolution maps. Conversely, district level maps only were produced in the following cases: i) where indicators or rates were derived through application of a model to the household survey data; ii) for indicators classified as rare events; iii) where input data sources were already at district level and no finer scale resolutions were available. For cases i) and ii) we define the data produced as maps of rare events or model-based indicators at district level, and the main source of data was the NFHS-4. All datasets were finally harmonised and aggregated at district level. Figure 1 shows a flowchart of the data preparation and processing methods adopted to generate gridded and district level reproductive, maternal, newborn, child, and adolescent health and development indicators datasets in India. Details of each indicator including definition, geographical level of aggregation of the output dataset, data source and year are outlined in Tables1,2.

**Table 1:** Indicators’ names, definitions, sources and year for 5 km × 5 km high-resolution reproductive, maternal, newborn, child, and adolescent health and development indicators for India.

Name	Definition	Geographical level	Source	Year
Low birth weight	Percentage of live births in the five (or three) years preceding the survey whose birth weight is less than 2.5 Kg.	5 km × 5 km high- resolution	NFHS-4	2015–16
Use of contraception	Percentage (%) of currently married or in union women currently using any modern method of contraception.	5 km × 5 km high- resolution	NFHS-4	2015–16
Number of antenatal care visits	Percentage (%) of women who had a live birth in the five years preceding the survey who had 4+ antenatal care visits.	5 km × 5 km high- resolution	NFHS-4	2015–16
Timing of antenatal care visits	Percentage (%) of women who had a live birth in the five years preceding the survey whose first antenatal care visit was at less than 4 months.	5 km × 5 km high- resolution	NFHS-4	2015–16
Urine sample taken during antenatal care visit	The percentage (%) of women with a live birth in the five years preceding the survey who received antenatal care (ANC) for the most recent birth with urine sample taken.	5 km × 5 km high- resolution	NFHS-4	2015–16
Blood sample taken during antenatal care visit	The percentage (%) of women with a live birth in the five years preceding the survey who received antenatal care (ANC) for the most recent birth with blood sample taken.	5 km × 5 km high- resolution	NFHS-4	2015–16
Iron tablets or syrup received during antenatal care visit	The percentage (%) of women with a live birth in the five years preceding the survey who received iron tablets or syrup during antenatal care.	5 km × 5 km high- resolution	NFHS-4	2015–16
Children stunting	Percentage (%) of children under age five years stunted (below –2 SD of height-for-age according to the World Health Organisation’s (WHO) standard).	5 km × 5 km high- resolution	NFHS-4	2015–16
Children wasting	Percentage (%) of children wasted (below -2 SD of weight for height according to the WHO’s standard).	5 km × 5 km high- resolution	NFHS-4	2015–16
Female population with completed secondary education	Percentage (%) of women aged 15 to 49 who have completed secondary education at the time of the survey.	District level	IHME	2010–2015–2017
Net attendance rate for secondary school (girls)	Percentage (%) of secondary school age girls attending secondary school.	5 km × 5 km high- resolution	NFHS-4	2015–16
Net attendance rate for secondary school (boys)	Percentage (%) of secondary school age boys attending secondary school.	5 km × 5 km high- resolution	NFHS-4	2015–16
Child marriage (15 years old)	Percentage of women whose first marriage or consensual union occurred before the age of 15 over the full sample of women aged 15–49.	5 km × 5 km high- resolution	NFHS-4	2015–16
Child marriage (18 years old)	Percentage of women whose first marriage or consensual union occurred before the age of 18 over the full sample of women aged 15–49.	5 km × 5 km high- resolution	NFHS-4	2015–16
Female labour force participation	Percentage (%) of employed women among those currently in a union. Employment status in the last 12 months among those currently in a union. The indicator includes those who worked in the past year, those who are currently working and those who have a job but were on leave over the last 7 days.	5 km × 5 km high- resolution	NFHS-4	2015–16
Experience of physical violence	Percentage (%) of women aged 15–49 who have experienced physical violence since the age of 15 by anyone.	5 km × 5 km high- resolution	NFHS-4	2015–16
Women decision-making on her own health	Percentage (%) of married women who decide on own health care either alone or jointly with partner.	5 km × 5 km high- resolution	NFHS-4	2015–16
Children receiving vitamin A supplements	Percentage (%) of children aged 6–59 months who were given vitamin A supplements.	5 km × 5 km high- resolution	NFHS-4	2015–16
Comprehensive knowledge of HIV	Percentage (%) of women who have comprehensive knowledge of HIV. Comprehensive knowledge is defined as: knowing that consistent use of condoms during sexual intercourse and having just one uninfected faithful partner can reduce the chances of getting HIV/AIDS, knowing that a healthy-looking person can have HIV/AIDS, and rejecting two common misconceptions about transmission or prevention of HIV/AIDS.	5 km × 5 km high- resolution	NFHS-4	2015–16

### Data collection, preparation, and processing

#### Estimating 5 km × 5 km high resolution datasets using cluster level proportions from household surveys

##### India NFHS-4: Geolocated and sub-nationally representative household survey

The 2015–16 India National Family Health Survey (NFHS-4) was conducted by the Ministry of Health and Family Welfare, Government of India and International Institute for Population Sciences, Mumbai, with the technical assistance of ICF through the Demographic and Health Surveys (DHS) Program (funded by USAID). NFHS-4 provides estimates of fertility, mortality, family planning, reproductive, maternal and child health, wealth and nutrition indicators at the national and state levels. Most of the indicators are also provided for the 640 districts of India (as per the Census, 2011)^22^.

NFHS-4 is based on a two-stage stratified sample of households, where 28,586 primary sampling units (PSUs), also called enumeration areas (EAs) or clusters, were first selected with probability proportional to the EA size and by urban and rural areas, with a total of 28,522 PSUs completed. The 2011 census served as the sampling frame for the selection of PSUs, where PSUs were villages in rural areas and Census Enumeration Blocks in urban areas. PSUs with fewer than 40 households were linked to the nearest PSU^22^. This first stage of selection provided a listing of households for the second stage, where segments of PSUs of approximately 100–150 households were randomly selected for the survey using systematic sampling with probability proportional to segment size. Survey clusters can therefore be either PSUs or segments of PSUs. Subsequently, in every selected rural and urban cluster, 22 households were randomly selected with systematic sampling, to create statistically reliable estimates of key demographic and health variables^29,30^. PSUs or EAs are usually pre-existing geographical areas which are derived from census. The boundaries of the EAs are defined by the country’s census bureau, as are the urban and rural status of each cluster. In recent DHS surveys geolocations (latitude and longitude) for each survey cluster are available. The survey cluster coordinates represent an estimated centre of the cluster and are collected in the field through GPS receivers. The georeferenced datasets can be linked to individual and household records in DHS household surveys through unique cluster identifiers. To protect the confidentiality of respondents, cluster locations are displaced up to 5 km in rural areas and up to 2 km in urban areas at the processing stage. A further 1% of the rural clusters can be displaced up to 10 km. Because displacement affects the physical location of the data, it is necessary to account for displacement when undertaking spatial modelling with DHS surveys^31,32^.

### Construction of the indicators for high resolution mapping using NFHS-4

Cluster-level proportions of reproductive, maternal, newborn, child, and adolescent health and development indicators were calculated and used as input data to construct 5 km × 5 km gridded high resolution maps using geospatial modelling techniques, where the GPS from the surveys and spatial covariates were exploited to predict surfaces^33–36^.

The construction of cluster level indicators from the India NFHS-4 survey followed the definitions and instructions of the DHS programme^22,37,38^. Details of each indicator are outlined in Tables 1 and 2.

### Geospatial covariates for high resolution mapping

We considered variables that are known to influence or are proxies for other variables that are known to influence the health and development indicators in this study. We categorized them as geographical, socioeconomic, and environmental variables; see Table SI.1. We also called these variables “geospatial covariates”. Geospatial covariates are important for model construction, parameter estimation and prediction. They provide information on the observed spatial distribution of the response variables and are utilized as predictors to improve the predictions of the response variables^28,35,39^. Since the geospatial covariates were collated from different sources, we adjusted them such that they are all gridded datasets at the 1 km × 1 km resolution. For modelling purposes, we aggregated the geospatial covariate gridded datasets further to a 5 km × 5 km resolution. The geospatial covariates at each health and development surveyed cluster location were extracted using ESRI ArcGIS v10.6.

### Constructing high resolution maps for indicators with geospatial modelling techniques

**Fig. 2:**
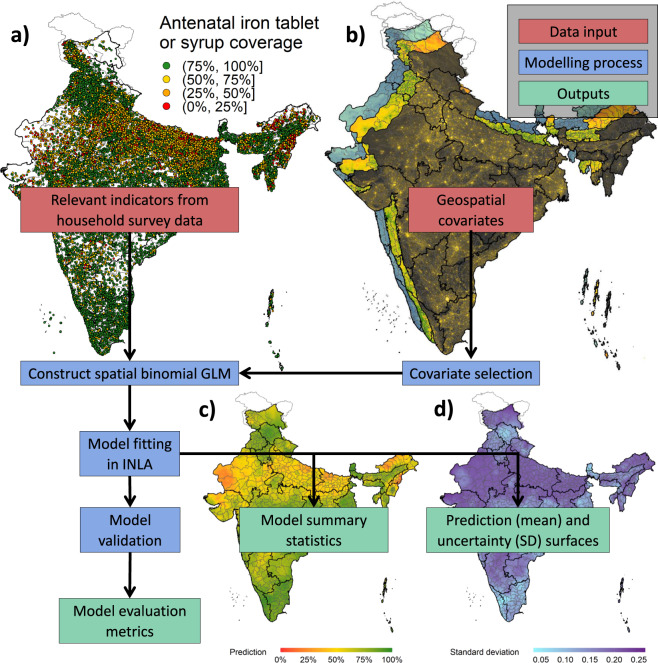
Flow chart outlining the model constructing, fitting, and validating process of the health and development indicators. (**a**) The DHS geolocated household survey dataset of iron tablets or syrup received during antenatal care visits. (**b**) Geospatial covariates stack at the 1 × 1 km resolution. (**c**) Prediction (mean) surface for antenatal iron or syrup coverage at the 5 km × 5 km resolution. (**d**) Uncertainty (standard deviation) for antenatal iron or syrup coverage at the 5 km × 5 km resolution.

To construct prediction and uncertainty surfaces for the health and development indicators, we used the following: the health and development indicator datasets, the geospatial covariate gridded datasets, and the boundary information. The methodology involved constructing models, fitting the models, prediction with the models and validating the models; see Fig. 2 for an illustration of the workflow.

The model construction was two-staged. In the first stage, we checked for multicollinearity amongst the geospatial covariates. In the second stage, we employed the backward stepwise model selection algorithm to select the optimal set of geospatial covariates for the target indicator.

To check for multicollinearity, we first created a Pearson correlation matrix for the geospatial covariates and any pairs with a Pearson correlation coefficient r > 0.8 were flagged. The flagged covariates were then individually fitted in non-Bayesian binomial generalised linear models (GLMs). We then calculated the Bayesian information criteria (BIC) of the models. The covariate in the model with a lower BIC was retained while the covariate in the model with the greater BIC was omitted. To further ensure that multicollinearity between the remaining geospatial covariates was not present, we calculated the variance inflation factors (VIFs) and any covariate that had a VIF > 4 was omitted.

After checking for multicollinearity, a backward model selection algorithm was used to select the best subset of geospatial covariates for the target indicator. To obtain the optimal set of geospatial covariates, the following steps were followed:

1. The remaining geospatial covariates were fitted in a non-Bayesian binomial GLM and the BIC was calculated.
2. A covariate was removed from the fitted model and the BIC recalculated.
3. If the recalculated BIC was less than the previously calculated BIC, this subset of covariates was preferred.
4. These steps were performed iteratively until the recalculated BIC is not less than the BIC calculated from the previous iteration.

Using the optimal set of geospatial covariates obtained and each health and development indicator as input data, a Bayesian point-referenced spatial binomial GLM fitted in INLA was fitted.

For *i* = 1,…,*n*, let *Y*(***s***_*i*_) denote the number of events of the target indicator at the survey cluster location ***s***_*i*_. For example, *Y*(***s***_*i*_) may be the number of women who use modern contraception or may be the number of women who received iron tablets or syrup during antenatal care visits; see Tables 1,2 for the full list of health development indicators considered in this study. Furthermore, let *m*(***s***_*i*_) denote the total number of surveys conducted within the survey cluster location. The Bayesian point-referenced spatial binomial GLM is given as follows:

1 $$\begin{array}{c}Y({{\boldsymbol{s}}}_{i})|{\boldsymbol{m}}({{\boldsymbol{s}}}_{i})\sim {\rm{B}}{\rm{i}}{\rm{n}}{\rm{o}}{\rm{m}}{\rm{i}}{\rm{a}}{\rm{l}}({\boldsymbol{m}}({{\boldsymbol{s}}}_{i}),{\boldsymbol{p}}({{\boldsymbol{s}}}_{i})),\\ {\rm{l}}{\rm{o}}{\rm{g}}{\rm{i}}{\rm{t}}({\boldsymbol{p}}({{\boldsymbol{s}}}_{i}))={\bf{x}}{\prime} ({{\boldsymbol{s}}}_{i}){\boldsymbol{\beta }}+{\boldsymbol{\omega }}({{\boldsymbol{s}}}_{i})+\epsilon ({{\boldsymbol{s}}}_{i}).\end{array}$$

2 $${\boldsymbol{\omega }}\left({{\boldsymbol{s}}}_{i}\right) \sim {N}_{n}\left(0,{\Sigma }_{\omega }\right),$$

3 $${\Sigma }_{\omega }={\sigma }_{\omega }^{2}\exp \left(-\phi D\right).$$

$$\epsilon \left({{\boldsymbol{s}}}_{i}\right) \sim N\left(0,{\sigma }_{\epsilon }^{2}\right)$$

**Table 2:** Indicators’ names, definitions, sources and year for district level reproductive, maternal, newborn, child, and adolescent health and development indicators for India.

Name	Definition	Geographical level	Source	Year
Stillbirths	Number of pregnancies that lasted seven or more months and terminated in a foetal death in the five years preceding the survey per 1000 births (stillbirths plus the number of live births in the five years preceding the survey).	District level	NFHS-4	2015–16
Teenage pregnancies	Percentage (%) of women within 15–19 years old who have given birth or are pregnant with their first child over the full sample of women aged 15–49.	District level	NFHS-4	2015–16
Child mortality rate	The probability (expressed per 1000 children surviving their first birthday) of a child dying on or after their first birthday but before reaching the age of five years over a 5-year reference period.	District level	NFHS-4	2015–16
Total fertility rate	Total fertility rate for the three years preceding the survey for age group 15–49 expressed per woman.	District level	NFHS-4	2015–16
Total fertility rate for age group 15–19	Age-specific fertility rate for the three years preceding the survey for age group 15–19 expressed per 1,000 women.	District level	NFHS-4	2015–16
Total fertility rate for age group 20–24	Age-specific fertility rate for the three years preceding the survey for age group 20–24 expressed per 1,000 women.	District level	NFHS-4	2015–16
Neonatal mortality rate	The probability (expressed per 1000 live births) of a child dying before reaching the age of 1 month over a 5-year reference period.	District level	NFHS-4	2015–16
Night time lights	Satellite-derived night time lights have been used as a proxy to measure energy consumption. Nightlight intensity data displaying radiance measured as nanoWatts/cm2/sr.	District level	WorldPop/NOAA	2016
Air quality	Particulate matter PM2.5 concentration - 10 μg/m3 is WHO’s threshold above which health impacts become more severe.	District level	Socioeconomic Data and Applications Centre (SEDAC)	2016

*Y*(***s***_*i*_) follows a Binomial distribution with the parameter *p*(***s***_*i*_) which denotes the proportion of events happening at the survey cluster ***s***_*i*_. Following the examples above, this may be the proportion of women who use modern contraception or the proportion of women who received iron tablets or syrup during antenatal care visits. The model then assumes a logit link on *p*(***s***_*i*_) with the linear predictors which consist of the fixed effects **x**ʹ(***s***_*i*_)**β**, spatial random effects *ω*(***s***_*i*_) and independent identical (iid) random effects *ϵ*(***s***_*i*_) as shown in Eq. (1).

The fixed effects are given by the geospatial covariates **x**ʹ(***s***_*i*_) selected from the backward model selection algorithm mentioned above and ***β*** is a vector of regression coefficients to be estimated. The spatial random effects follow a multivariate normal distribution with zero-mean and some covariance matrix Σ_*ω*_ as shown in Eq. (2). In this study, elements of the covariance matrix are calculated with the exponential covariance function as shown in Eq. (3). The exponential covariance function is calculated with the spatial variance ${\sigma }_{\omega }^{2}$, the spatial decay parameter *ϕ* and the *n* × *n* Euclidean distance matrix *D* between the survey cluster locations. The parameters ${\sigma }_{\omega }^{2}$ and *ϕ* are unknown and are to be estimated in INLA. The iid random effects follow a normal distribution with a mean of zero and an unknown variance ${\sigma }_{\epsilon }^{2}$ which will be estimated along with the other parameters mentioned above.

We estimated the parameters of Eq. (1) in the Bayesian framework with the integrated nested Laplace approximation (INLA) method in conjunction with the stochastic partial differential equation (SPDE) approach^40,41^. More specifically, the parameters are the regression coefficients ***β***, the spatial range (3/*ϕ*), the variance of the spatial random effect ${\sigma }_{\omega }^{2}$, and the variance of the iid random effect ${\sigma }_{\epsilon }^{2}$. The INLA method was developed by Rue *et al*.^41^ as an alternative to the traditional Markov Chain Monte Carlo methods used for modelling and parameter estimation in the Bayesian framework. It reduces computation time through analytic approximations with the Laplace method; see^41,42^ for a more comprehensive commentary on INLA. The SPDE approach projects continuous Gaussian fields, such as Eq. (2), as discrete Gaussian Markov random fields to further reduce computation cost^43^. This projected surface is called a “mesh” since the projection involves triangulating the spatial domain under consideration. The decision lies in the number of triangles to create within the spatial domain as more triangles will improve the approximation but increases computation time^44^. The SPDE approach implemented with the INLA method is useful for high dimensional problems such as parameter estimation for spatial models. The INLA method can be implemented in the statistical programming software R^45^ with the INLA package^40,41^.

To fit Eq. (1) with the INLA-SPDE approach, we specify non-informative priors *N*(0,1000) on ***β***, *LogGamma*(2,1) on the variance of the iid random effect, and penalized complexity^46^ priors $p\left({r}_{sp} < {r}_{0}\right)=0.01$ and $p\left({\sigma }_{\omega } < 3\right)=0.01$ for (3/*ϕ*) and ${\sigma }_{\omega }^{2}$ respectively. Here, *r*_*sp*_ denotes the spatial range to avoid confusion with the Pearson correlation coefficient and *r*_0_ is calculated as 5% of the extent of India in the east-west direction. The parameter *σ*_*ω*_ is called the partial sill and is the square-root of ${\sigma }_{\omega }^{2}$. The mesh was constructed by supplying the coordinates of the surveyed clusters and additional arguments to determine the number of triangles to construct within our study domain.

After fitting the model with INLA, we predict using the geospatial covariate gridded datasets at the 5 km × 5 km resolution. We extract the mean and the standard deviation from the distribution of the prediction at each grid to create the prediction and uncertainty surfaces.

An application of the modelling framework just described showing how to construct 5 km × 5 km high resolution map and uncertainty for the percentage of women who received iron tablets or syrup during antenatal care visits is presented in SI.2 to SI.6.

Tables SI.7 show the summary statistics of the fitted models for each health and development indicator calculated at 5 km × 5 km high-resolution using INLA.

#### Estimating district level proportions and rates from household surveys for other indicators based on NFHS-4

##### Construction of indicators at district level using NFHS-4

The India NFHS-4 survey was constructed to be representative at national, province and district level for most of the indicators. In the case of rare events indicators such as stillbirth rates, or where more sophisticated estimation methods were needed such as mortality rates, indicators were constructed and mapped at district level (denoted in this work as “NFHS-4 rare events indicators or model-based district level indicators”). Mortality rates and the fertility rates were modelled using a generalised linear model and consider the number of occurrences (birth or deaths) as a random variable^47^. The distribution of the random variable of occurrences is assumed to be Poisson in the case of fertility rates and binomial for mortality rates. The child mortality rate was calculated using a synthetic cohort life table approach which combines mortality probabilities for specific age segments (12–23, 24–35, 36–47, and 48–59) into the standard age segment (1 to 4 years). Given the scarcity of occurrences measuring the events of interest across small-scale geographical areas (i.e., clusters) district level estimates were created. For example, for stillbirth rates the amount of cluster with no data was around 90% while for teenage pregnancies it was about 75%.

The confidence intervals for modelled rates, mortality, and fertility rates were calculated using the delta method to estimate the standard error using the variance-covariance matrix of the modelled rates^47^. The confidence intervals for proportions (e.g., teenage pregnancies) were calculated using the Wilson Score method^48–51^. The confidence intervals for the stillbirth rate were calculated using Byar’s approximation for counts above 5^52–54^ while tables of the exact probabilities were used for counts below 5^55^.

The construction of district level indicators from the India NFHS-4 survey followed the definitions and instructions of the DHS programme^22,37,38^. Details of each indicator are outlined below in Tables 1,2.

#### District or State level data not available through household surveys or already estimated

For indicators where data was not available in the NFHS-4, we used data from other openly available data sources, these included: the Socioeconomic Data and Applications Centre (SEDAC) https://sedac.ciesin.columbia.edu/^56,57^, from which we derived Global Annual PM2.5 Grids for years 2000–2015, satellite-derived night-time lights processed by WorldPop (2016)^58,59^ which was used as a proxy for energy consumption and, Institute for Health Metrics and Evaluation (IHME) http://www.healthdata.org/, used to obtain the data on women aged 15 to 49 who have completed secondary education for 2010, 2015 and 2017^60^.

#### Summarization at the district level and joining to boundaries

The data for 28 health and development indicators including high-resolution estimates and district level estimates were matched and summarised using an adapted vector geographical boundary (shapefile), based on the 2011 census, obtained from DataMeet Community Maps Project^61^.

**Fig. 3:**
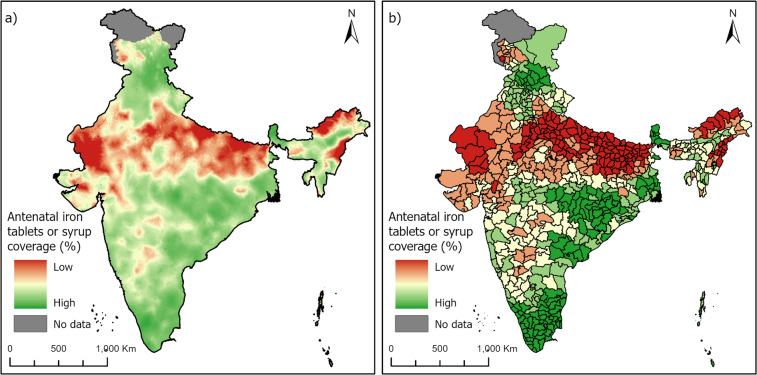
5 km × 5 km high-resolution (panel a) and district level summary (mean) (panel b) for the percentage of women receiving iron tablets or syrup during ANC visits.

Figure 3 below shows an example of an indicator at 5 km × 5 km high resolution (left panel) and summarised at the district level (right panel) for the percentage of women receiving iron Tablets or syrup during ANC visits. Indicator at high resolution allow users to summarise the data to a custom based area, while district area allows the comparability of the data at a known administrative level.

## Data Records

The different types of data available described in this article referring to India are listed in Table 3. The high-resolution maps of the modelled indicators with their associated uncertainty have been compiled^62^. All the indicators estimated in this article have been summarised at the district level and have been compiled in a shapefile and CSV^63^ for those rare events/ model-based indicators confidence intervals were calculated and compiled at the district level in a shapefile and a CSV^64^.

**Table 3:** Name and description of datafiles on available indicators in India.

Name	Description	Resolution	Files Format	University of Southampton DOI
High resolution gridded datasets with estimates and uncertainties for health and development indicators in India	High resolution gridded datasets with estimates and uncertainties for health and development indicators in India	5 km × 5 km	GeoTIFF	10.5258/SOTON/WP00738
District level dataset with estimates for health and development indicators in India	-Summarised estimates of high-resolution data aggregated at district level;-District level estimates for rare events indicators/ model-based from NFHS-4;-District level estimates for other sources for India	District	shapefile	10.5258/SOTON/WP00739
District level confidence intervals for rare events/modelled-based indicators in India	District level confidence intervals for rate events/model-based indicators from NFHS-4, including the difference between upper and lower limit of the 95% confidence interval relative to the point estimator of each indicator	District	shapefile	10.5258/SOTON/WP00740

The input data used to produce this work are freely available after approval of registration and with a signed data access agreement on the websites of the data providers (i.e., NFHS-4). All other data sources were openly available and are referenced in Table SI.1.

## Technical Validation

### Model validation for the bayesian point-referenced spatial binomial GLM model used to construct high resolution maps

To access the performance of the model constructed for the target indicator, we used the *k*-fold cross validation and computed several evaluation metrics. The *k*-fold cross validation partitions the dataset into *k* parts then trains the model with *k*-1 parts of the dataset and tests the trained model with the *k*th part of the dataset. We calculated the following evaluation metrics:

4 $$\rho \left(\widehat{{\bf{p}}},{\bf{p}}\right),$$

5 $$\sqrt{\frac{1}{{n}_{{\rm{test}}}}{\sum }_{i=1}^{{n}_{{\rm{test}}}}{\left({\widehat{p}}_{i}-{p}_{i}\right)}^{2}},$$

6 $$\frac{1}{{n}_{{\rm{test}}}}{\sum }_{i=1}^{{n}_{{\rm{test}}}}\left|{\widehat{p}}_{i}-{p}_{i}\right|,$$

7 $$\left(\frac{{\sum }_{i=1}^{{n}_{{\rm{test}}}}\left({\widehat{p}}_{i}-{p}_{i}\right)}{{\sum }_{j=1}^{{n}_{{\rm{test}}}}\left({p}_{j}\right)}\right)\times 100.$$

the Pearson’s correlation coefficient, the root mean squared error, the mean absolute error, and the percentage bias. In the evaluate metrics above, *p*_*i*_ is used to denote the observed values – i.e., the proportions of the target indicators partitioned for testing – and ${\widehat{p}}_{i}$ is used to denote the predicted mean values from the Bayesian point-referenced spatial binomial GLM.

The notation *ρ*(⋅) is used to the denote the Pearson’s correlation coefficient in Eq. (4). Explicitly this is calculated with the covariance of the observed and predicted values and the standard deviation of the observed and predicted values

$$\rho \left(\widehat{{\bf{p}}},{\bf{p}}\right)=\frac{{\rm{cov}}(\widehat{{\bf{p}}},{\bf{p}})}{{\sigma }_{\widehat{{\bf{p}}}}{\sigma }_{{\bf{p}}}}$$

Here, note that the vectors $\widehat{{\boldsymbol{p}}}=\left({\widehat{p}}_{1},\ldots ,{\widehat{p}}_{{n}_{test}}\right)$ and ${\boldsymbol{p}}=\left({p}_{1},\ldots ,{p}_{{n}_{test}}\right)$ where *n*_*test*_ is the number of observations partitioned for testing. Better predictive performance is reflected from a greater Pearson’s correlation coefficient. The root mean squared error (RMSE), mean absolute error (MAE) and percentage bias is given in Eqs. (5–7) respectively. Better predictive performance is reflected from smaller RMSE, MAE and percentage bias values.

Table SI.8 show the summary of model validation metrics for each health and development indicator calculated at 5 km × 5 km high-resolution using INLA.

### Confidence intervals for estimates of district level indicators calculated using NFHS-4

For those indicators where NFHS-4 district level estimates were produced (rare events and model-based district level indicators), we provided raster data of uncertainty associated with the indicators by mapping the difference between upper and lower limit of the 95% confidence interval relative to the point estimator of the indicator. The narrower the confidence interval, i.e., the smaller the value, lesser the uncertainty around the estimated indicator and thus higher the precision. More information on how confidence intervals were constructed can be found in the section “Construction of indicators at district level using NFHS-4”.

### Accuracy of data

The accuracy and quality of estimates from survey data such as those provided by the DHS (NFHS) have been assessed in several reports outside this work^65,66^. Input data (e.g. survey clusters and covariates) carry some degree of uncertainty which may affect the actual values in small areas. In particular, the low birth weight indicator has a low degree of correlation (see SI.8) and the quality of the birthweight data from the DHS surveys has been widely investigated. The authors recommend using the birthweight indicator with caution^67–69^. Authors recommend to use the birthweight indicator with caution. The introduction of cluster location random displacement can introduce uncertainty although in general studies have shown that the impact of displacement is considered to be limited^70,71^. Other sources of uncertainty may also be due to temporal miss-match of some of the covariates, as discussed in previous works^72,73^.

Most of the data used in this work, and in particular NFHS-4 round, refer to years 2015–16. At the time of writing, NFHS-4 round was the latest available survey for India. Upcoming work will focus on constructing a similar atlas using the new NFHS-5 data just released and assessing changes between the round 4 and 5.

## Usage Notes

The datasets presented here can be used both to (i) support applications measuring sub-national metrics of reproductive, maternal, newborn, child, and adolescent health and development for India and (ii) to inform planning decisions, target interventions and development programs. However, considering that the gridded high-resolution datasets represent modelling outputs generated using ancillary covariates, to avoid circularity, they should not be used to make predictions or explore relationships about any of those ancillary datasets^74^. Thus, before using the gridded high-resolution datasets in correlation analyses against factors which are included in their construction (e.g., correlating children stunting with temperature), ideally the modelling process should be re-run using the code provided with this work^75^, with the applicable covariates removed.

Moreover, when using estimates produced as a result of a modelled output, a degree of uncertainty always needs to be taken into account. Please, refer to the uncertainty data which were produced in the context of this work.

## Supplementary information

Supplementary Information

## Data Availability

The code for modelling, prediction and validation is publicly available via the project GitHub repository^75^. The code was written and ran in R version 4.0.4, and it is dependent on the R package INLA. Further documentation regarding the scripts can be found in the README file within the GitHub repository. Instructions and code for constructing reproductive, maternal, newborn, child, and adolescent health and development indicators using NFHS surveys and DHS data which were used as input data can be found on the DHS Programme GitHub repository (www.github.com/DHSProgram).
