# Supplementary material for: A subnational reproductive, maternal, newborn, child, and adolescent health and development atlas of India
Source: Sci Data. 2023 Feb 10;10:86. doi: 10.1038/s41597-023-01961-2 (PMC9918481; doi:10.1038/s41597-023-01961-2)
Supplement: Supplementary file 1 — Supplementary Information [file 41597_2023_1961_MOESM1_ESM.docx]

**Supplementary Information**

**A subnational** **reproductive, maternal, newborn, child, and adolescent health and development atlas of India.**

Carla Pezzulo^1^, Natalia Tejedor-Garavito^1^, Ho Man Theophilus Chan^1-2^, Ilda Dreoni^1-3^, David Kerr^1^, Samik Ghosh ^4^, Amy Bonnie^1^, Maksym Bondarenko^1^, Mihretab Salasibew^4^, Andrew J. Tatem^1^

^1^WorldPop, School of Geography and Environmental Science, University of Southampton, Highfield Campus, Southampton, SO17 1BJ, UK

^2^School of Mathematical Sciences, University of Southampton, Southampton, UK

^3^Social Statistics & Demography, University of Southampton, Highfield Campus, Southampton, SO17 1BJ, UK

^4^Children's Investment Fund Foundation (CIFF), London, UK

**Table Of Contents**

**Table SI.1.** Table of geospatial covariates, and their description, assembled for testing when modelling the health and development indicators……………………………………………………………………Page 2

**SI.2.** An application: producing 5kmx5km high resolution map and uncertainty for the percentage of women who received iron tablets or syrup during antenatal care visits……………………………………Page 4

**Table SI.3.** Summary statistics of the fitted model for the indicator iron tablets or syrup received during antenatal care visits……………………………………………………………………………………………………….Page 5

**Table SI.4.** Model validation summary of the fitted model for the indicator iron tablets or syrup received during antenatal care visits..……………………………………………………………………………………….Page 5

**SI.5** Prediction map of the percentage of women who received iron tablets or syrup during antenatal care visits………………………………………………………………………………………………………………………………….Page 6

**SI.6** Uncertainty map of the percentage of women who received iron tablets or syrup during antenatal care visits…………………………………………………………………………………………………………………..Page 7

**SI.7.** Summary statistics of the fitted models for each health and development indicator calculated at 5kmx5km high-resolution using INLA………………………………………………………………………………………..Page 8

**SI.8.** Summary of model validation metrics for each health and development indicator calculated at 5kmx5km high-resolution using INLA…………………………………………………………………………………….Page 13

**Table SI.1.** Table of geospatial covariates, and their description, assembled for testing when modelling the health and development indicators. All geospatial covariates tested were continuous.

| **Category** | **Name** | **Description** | **Year** | **Source** |
| --- | --- | --- | --- | --- |
| Geographical covariates | Distance to major roads | Distance to major OpenStreetMap roads (km) | 2016 | *[Derived from]* Open Street Map (2019). OpenStreetMap Contributors [www.openstreetmap.org](http://www.openstreetmap.org/) |
|  | Distance to protected areas | Distance to protected areas as defined by the World Database on protected areas (km) | 2016 | *[Derived from]* Open Street Map (2019). OpenStreetMap Contributors [www.openstreetmap.org](http://www.openstreetmap.org/) |
|  | Distance to waterways | Distance to OpenStreetMap waterways (km) | 2016 | *[Derived from]* Open Street Map (2019). OpenStreetMap Contributors [www.openstreetmap.org](http://www.openstreetmap.org/) |
|  | Elevation | Digital elevation model (m) | 2001 | de Ferranti, J. (2017). 'Digital Elevation Data'. [Viewfinder Panoramas](http://www.viewfinderpanoramas.org/dem3.html); based on NASA's Shuttle Radar Topography Mission ([SRTM](http://www2.jpl.nasa.gov/srtm/))) data. |
|  | Slope | Shuttle Radar Topography Mission (SRTM) slope (degrees) | 2001 | de Ferranti, J. (2017). 'Digital Elevation Data'. [Viewfinder Panoramas](http://www.viewfinderpanoramas.org/dem3.html); based on NASA's Shuttle Radar Topography Mission ([SRTM](http://www2.jpl.nasa.gov/srtm/))) data. |
|  | Travel time | Travel time to major cities (mins) | 2015 | Weiss, D.J. et al. (2018). A global map of travel time to cities to access inequalities in accessibility in 2015. Nature. |
| Socioeconomic covariates | Cattle density | Cattle density (no./km^2^) | 2010 | Gilbert, M. et al. (2018) Global Distribution Data for Cattle, Buffaloes, Horses, Sheep, Goats, Pigs, Chickens and Ducks in 2010. Nature Scientific data, 5:180227. doi: 10.1038/sdata.2018.227 |
|  | Net primary production | MODIS net primary production (kg C/ m^2^) | 2005 | Running et al. (2015) MOD17A3H MODIS/Terra Net Primary Production Yearly L4 Global 500 m SIN Grid V006 [Data set]. NASA EOSDIS Land Processes DAAC. |
|  | Nighttime lights | Nighttime lights (nano-watts) | 2016 | NOAA – Visible Infrared Imaging Radiometer Suite. <https://ngdc.noaa.gov/eog/viirs/index.html> |
| Environmental covariates | Aridity | Mean aridity index (as developed from CGIAR-CSI data) | 1950-2000 | CGIAR-CSI Global-Aridity and Global-PET Database. Available at <https://cgiarcsi.community/data/global-aridity-and-pet-database/> |
|  | Evapotranspiration | Average annual potential evapotranspiration (as developed from CGIAR-CSI data) | 1950-2000 | CGIAR-CSI Global-Aridity and Global-PET Database. Available at <https://cgiarcsi.community/data/global-aridity-and-pet-database/> |
|  | Precipitation | Precipitation (mm) | 1970-2000 | Fick, S. E., & Hijmans, R. J. (2017). WorldClim 2: new 1‐km spatial resolution climate surfaces for global land areas. *International journal of climatology*, *37*(12), 4302-4315. |
|  | Temperature | Temperature (degree Celsius x 10) | 2005 | Wan, Z. *et al*. MOD11C3 MODIS/Terra Land Surface Temperature/Emissivity Monthly L3 Global 0.05Deg CMG Voo6 [Data set]. NASA EOSDIS Land Processes DAAC. |

## **SI.2.** An application: producing 5kmx5km high resolution map and uncertainty for the percentage of women who received iron tablets or syrup during antenatal care visits.

We demonstrate the methodology described above with one of the health development indicators considered in this study named “The percentage of women who received iron tablets or syrup during antenatal care visits (ANC)”. For $i=1, \ldots, n$, let $Y(\mathbf{s}_{i})$ denote the number of women with a live birth in the five years preceding the survey who received iron tablets or syrup during antenatal care (ANC) visits at survey cluster $\mathbf{s}_{i}$. Let $m\left( \mathbf{s}_{i} \right)$ denote the total number of women surveyed at survey cluster $\mathbf{s}_{i}$. Let ${p\mathbf{(s}}_{i})$ denote the proportion of women with a live birth in the five years preceding the survey who received iron tablets or syrup during antenatal care visits at survey cluster $\mathbf{s}_{i}$. From the model selection process, we choose net primary production, temperature, distance to protected areas, nighttime lights, and precipitation as the geospatial covariates to model the target indicator. Estimates of the model parameters from INLA are shown in Table SI.3. The estimates show that all covariates were significant predictors except for distance to protected areas and precipitation. Net primary production is shown to have a negative effect on the target indicator, whereas temperature and nighttime lights have a positive effect on the proportion of women who received iron tablets or syrup during ANC visits. The spatial range (3/$\phi$), spatial variance $\sigma_{\omega}^{2}$ and iid variance $\sigma_{\epsilon}^{2}$ were also significant. The spatial range corresponds to around 341km which indicates spatial correlation residuals in the model. The prediction and uncertainty surfaces are available in the provided datasets and also shown in SI.5-6. Relatively lower predicted proportions of women who received iron tablets and syrup during ANC visits are observed in the northern regions of India. The cross-validation evaluation metrics are given in Table SI.4.

**Table SI.3.** Summary statistics of the fitted model for the indicator iron tablets or syrup received during antenatal care visits.

|  | **Mean** | **SD** | **2.5%** | **97.5%** |
| --- | --- | --- | --- | --- |
| (Intercept) | 1.200 | 0.484 | 0.262 | 2.162 |
| Net primary production | -0.112 | 0.020 | -0.151 | -0.073 |
| Temperature | 0.003 | 0.001 | 0.001 | 0.004 |
| log(distance to protected areas) | -0.009 | 0.007 | -0.023 | 0.006 |
| log(nighttime lights) | 0.178 | 0.011 | 0.157 | 0.200 |
| log(precipitation) | 0.094 | 0.090 | -0.083 | 0.269 |
| Spatial range (3/$\phi$) | 3.081 | 0.348 | 2.491 | 3.853 |
| Spatial variance ($\sigma_{\omega}^{2}$) | 1.116 | 0.139 | 0.880 | 1.424 |
| iid variance ($\sigma_{\epsilon}^{2}$) | 0.709 | 0.017 | 0.676 | 0.742 |

**Table SI.4.** Model validation summary of the fitted model for the indicator iron tablets or syrup received during antenatal care visits.

|  | **Correlation** | **RMSE** | **MAE** | **Bias** |
| --- | --- | --- | --- | --- |
| In-sample | 0.592 | 0.197 | 0.151 | 0.935 |
| Out-of-sample | 0.569 | 0.200 | 0.154 | 0.935 |

**SI.5** Prediction map of the percentage of women who received iron tablets or syrup during antenatal care visits.


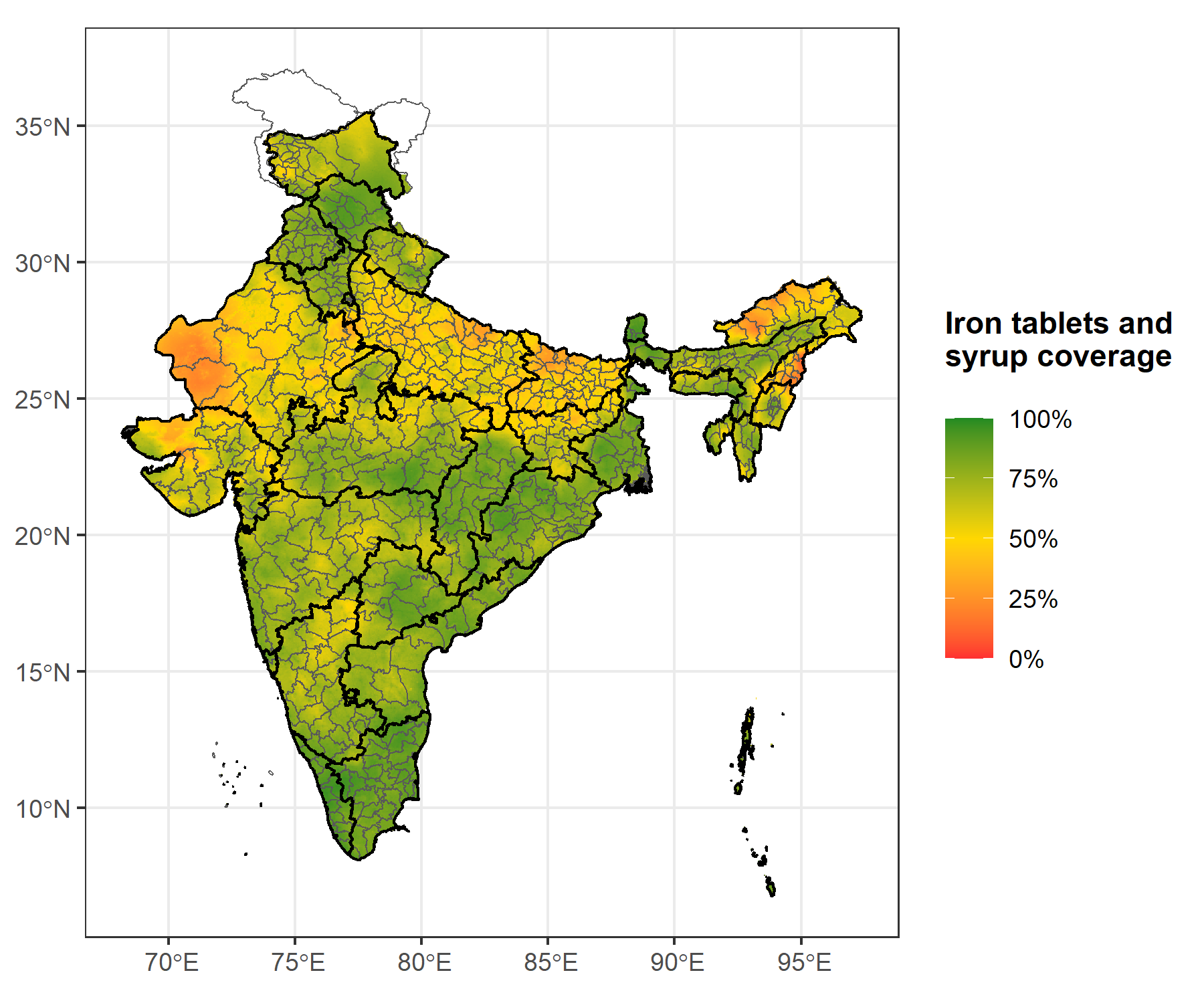


**SI.6.** Uncertainty map of the percentage of women who received iron tablets or syrup during antenatal care visits.


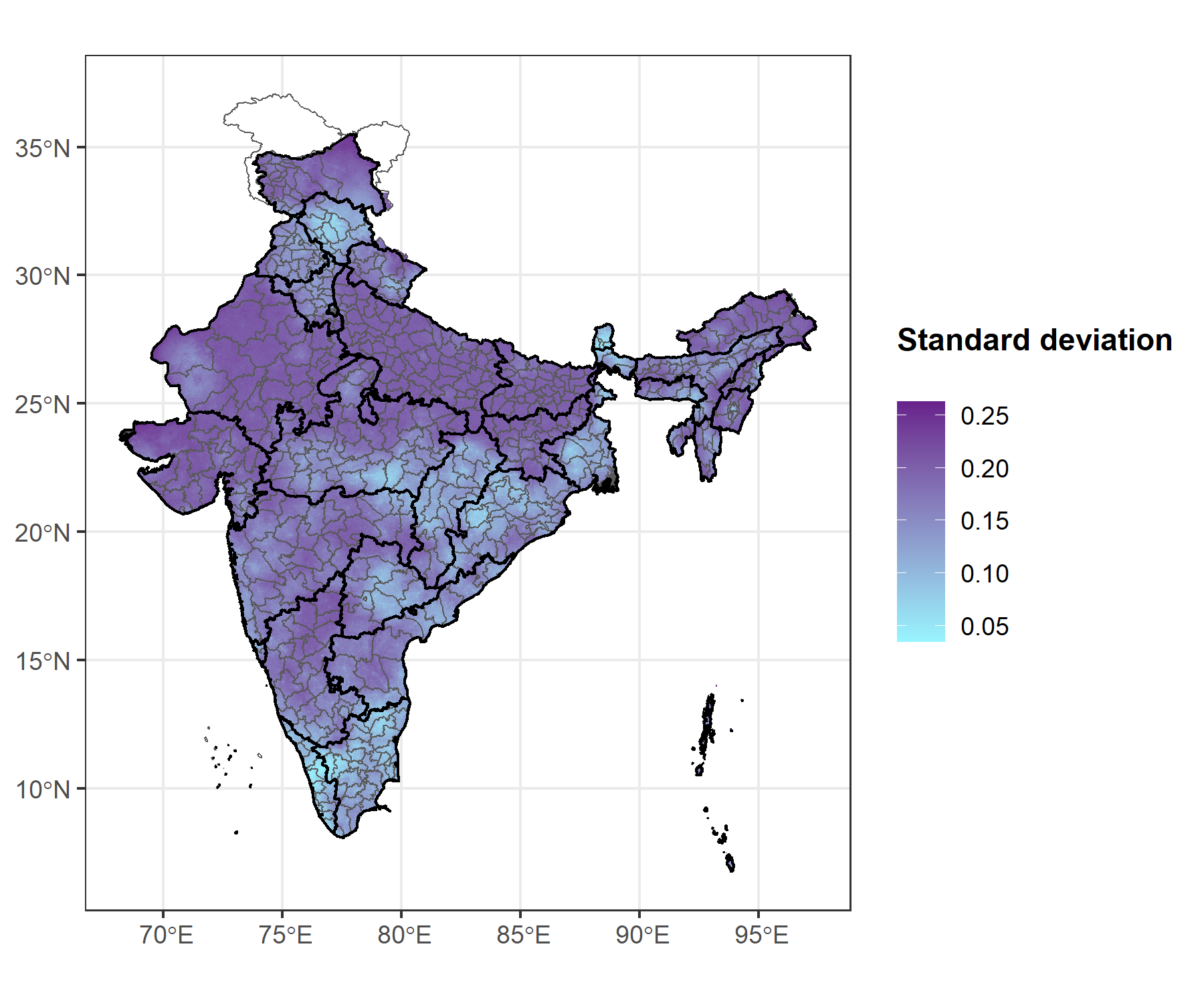


# SI.7. Summary statistics of the fitted models for each health and development indicator calculated at 5kmx5km high-resolution using INLA.

**Low birth weight**

|  | **Mean** | **SD** | **2.5%** | **97.5%** |
| --- | --- | --- | --- | --- |
| (Intercept) | -3.920 | 1.332 | -6.536 | -1.306 |
| Distance to highways | 0.001 | 0.003 | -0.004 | 0.006 |
| Net primary production | -0.046 | 0.015 | -0.076 | -0.016 |
| Aridity | -0.035 | 0.045 | -0.124 | 0.053 |
| log(nighttime lights) | 0.002 | 0.009 | -0.016 | 0.020 |
| log(evapotranspiration) | 0.308 | 0.180 | -0.045 | 0.660 |
| Spatial range (3/$\phi$) | 2.644 | 1.001 | 0.915 | 4.507 |
| Spatial variance ($\sigma_{\omega}^{2}$) | 0.151 | 0.023 | 0.110 | 0.199 |
| iid variance ($\sigma_{\epsilon}^{2}$) | 0.257 | 0.011 | 0.235 | 0.278 |

**Use of Contraception**

|  | **Mean** | **SD** | **2.5%** | **97.5%** |
| --- | --- | --- | --- | --- |
| (Intercept) | 3.894 | 4.101 | -4.173 | 12.003 |
| Slope | 0.001 | 0.004 | -0.007 | 0.009 |
| Distance to highways | 0.001 | 0.004 | -0.006 | 0.009 |
| Net primary production | -0.044 | 0.022 | -0.087 | 0.000 |
| Temperature | 0.004 | 0.002 | 0.001 | 0.008 |
| log(nighttime lights) | 0.014 | 0.011 | -0.008 | 0.036 |
| log(precipitation) | -0.293 | 0.105 | -0.494 | -0.085 |
| log(evapotranspiration) | -0.557 | 0.591 | -1.733 | 0.602 |
| Spatial range (3/$\phi$) | 5.176 | 1.954 | 1.971 | 9.434 |
| Spatial variance ($\sigma_{\omega}^{2}$) | 0.128 | 0.052 | 0.046 | 0.244 |
| iid variance ($\sigma_{\epsilon}^{2}$) | 0.214 | 0.007 | 0.200 | 0.228 |

**Number of antenatal care visits**  **(ANC 4+)**

|  | **Mean** | **SD** | **2.5%** | **97.5%** |
| --- | --- | --- | --- | --- |
| (Intercept) | 0.380 | 0.161 | 0.065 | 0.695 |
| Slope | -0.038 | 0.004 | -0.046 | -0.031 |
| Distance to highways | -0.016 | 0.004 | -0.023 | -0.008 |
| Distance to water sources | 0.005 | 0.002 | 0.002 | 0.009 |
| Net primary production | -0.013 | 0.020 | -0.052 | 0.027 |
| Aridity | 0.468 | 0.077 | 0.317 | 0.620 |
| log(nighttime lights) | 0.267 | 0.012 | 0.243 | 0.291 |
| Spatial range (3/$\phi$) | 1.925 | 0.089 | 1.777 | 2.125 |
| Spatial variance ($\sigma_{\omega}^{2}$) | 1.248 | 0.065 | 1.136 | 1.391 |
| iid variance ($\sigma_{\epsilon}^{2}$) | 1.06 | 0.018 | 1.022 | 1.092 |

**Timing of antenatal care visits**

|  | **Mean** | **SD** | **2.5%** | **97.5%** |
| --- | --- | --- | --- | --- |
| (Intercept) | 1.106 | 0.352 | 0.416 | 1.796 |
| Slope | -0.020 | 0.003 | -0.027 | -0.014 |
| Distance to highways | -0.018 | 0.003 | -0.025 | -0.012 |
| Distance to water sources | 0.006 | 0.002 | 0.003 | 0.009 |
| Net primary production | -0.040 | 0.018 | -0.075 | -0.004 |
| log(nighttime lights) | 0.183 | 0.011 | 0.162 | 0.204 |
| log(precipitation) | -0.033 | 0.075 | -0.180 | 0.114 |
| Spatial range (3/$\phi$) | 1.598 | 0.112 | 1.406 | 1.843 |
| Spatial variance ($\sigma_{\omega}^{2}$) | 0.897 | 0.073 | 0.776 | 1.062 |
| iid variance ($\sigma_{\epsilon}^{2}$) | 0.711 | 0.022 | 0.663 | 0.748 |

**Urine sample taken during antenatal care visit**

|  | **Mean** | **SD** | **2.5%** | **97.5%** |
| --- | --- | --- | --- | --- |
| (Intercept) | 17.748 | 9.559 | -0.983 | 36.546 |
| Slope | -0.027 | 0.008 | -0.043 | -0.010 |
| Distance to highways | -0.024 | 0.007 | -0.037 | -0.010 |
| Distance to water sources | 0.004 | 0.004 | -0.004 | 0.012 |
| Net primary production | 0.088 | 0.047 | -0.005 | 0.181 |
| Aridity | -0.157 | 0.161 | -0.473 | 0.159 |
| log(travel time to major cities) | -0.134 | 0.014 | -0.162 | -0.106 |
| log(distance to protected areas) | -0.008 | 0.015 | -0.038 | 0.020 |
| log(evapotranspiration) | -1.601 | 1.285 | -4.130 | 0.918 |
| Spatial range (3/$\phi$) | 5.020 | 0.712 | 3.849 | 6.633 |
| Spatial variance ($\sigma_{\omega}^{2}$) | 3.428 | 0.646 | 2.396 | 4.918 |
| iid variance ($\sigma_{\epsilon}^{2}$) | 0.267 | 0.036 | 0.202 | 0.344 |

**Blood sample taken during antenatal care visit**

|  | **Mean** | **SD** | **2.5%** | **97.5%** |
| --- | --- | --- | --- | --- |
| (Intercept) | 7.092 | 9.418 | -11.381 | 25.596 |
| Slope | -0.039 | 0.008 | -0.056 | -0.023 |
| Distance to highways | -0.017 | 0.007 | -0.032 | -0.003 |
| Distance to water sources | 0.009 | 0.004 | 0.000 | 0.018 |
| Net primary production | 0.163 | 0.043 | 0.079 | 0.246 |
| Aridity | -0.045 | 0.160 | -0.360 | 0.270 |
| log(distance to protected areas) | -0.017 | 0.015 | -0.047 | 0.011 |
| log(nighttime lights) | 0.278 | 0.024 | 0.231 | 0.324 |
| log(evapotranspiration) | -0.219 | 1.266 | -2.706 | 2.263 |
| Spatial range (3/$\phi$) | 4.982 | 0.756 | 3.692 | 6.657 |
| Spatial variance ($\sigma_{\omega}^{2}$) | 3.148 | 0.581 | 2.176 | 4.452 |
| iid variance ($\sigma_{\epsilon}^{2}$) | 0.205 | 0.03 | 0.148 | 0.267 |

**Children stunting**

|  | **Mean** | **SD** | **2.5%** | **97.5%** |
| --- | --- | --- | --- | --- |
| (Intercept) | -0.951 | 0.119 | -1.195 | -0.728 |
| Slope | 0.001 | 0.002 | -0.003 | 0.006 |
| Distance to highways | 0.006 | 0.002 | 0.002 | 0.01 |
| Net primary production | 0.006 | 0.012 | -0.018 | 0.031 |
| Aridity | -0.004 | 0.041 | -0.084 | 0.076 |
| log(distance to protected areas) | 0.002 | 0.005 | -0.007 | 0.011 |
| log(nighttime lights) | -0.105 | 0.007 | -0.120 | -0.091 |
| Spatial range (3/$\phi$) | 4.001 | 0.616 | 2.973 | 5.382 |
| Spatial variance ($\sigma_{\omega}^{2}$) | 0.186 | 0.027 | 0.141 | 0.248 |
| iid variance ($\sigma_{\epsilon}^{2}$) | 0.208 | 0.007 | 0.194 | 0.221 |

**Children wasting**

|  | **Mean** | **SD** | **2.5%** | **97.5%** |
| --- | --- | --- | --- | --- |
| (Intercept) | -10.018 | 1.435 | -12.816 | -7.190 |
| Slope | 0.011 | 0.003 | 0.006 | 0.017 |
| Distance to highways | 0.004 | 0.003 | -0.001 | 0.009 |
| Distance to water sources | -0.001 | 0.001 | -0.003 | 0.001 |
| Net primary production | 0.048 | 0.015 | 0.019 | 0.077 |
| log(nighttime lights) | -0.055 | 0.009 | -0.072 | -0.038 |
| log(evapotranspiration) | 1.138 | 0.195 | 0.753 | 1.518 |
| Spatial range (3/$\phi$) | 2.128 | 0.359 | 1.428 | 2.812 |
| Spatial variance ($\sigma_{\omega}^{2}$) | 0.194 | 0.020 | 0.158 | 0.236 |
| iid variance ($\sigma_{\epsilon}^{2}$) | 0.280 | 0.010 | 0.261 | 0.299 |

**Net attendance rate for secondary school (girls)**

|  | **Mean** | **SD** | **2.5%** | **97.5%** |
| --- | --- | --- | --- | --- |
| (Intercept) | 1.166 | 0.177 | 0.834 | 1.532 |
| Slope | -0.012 | 0.003 | -0.018 | -0.005 |
| Distance to highways | -0.014 | 0.003 | -0.019 | -0.008 |
| Distance to water sources | 0.001 | 0.001 | -0.001 | 0.004 |
| Net primary production | -0.035 | 0.016 | -0.065 | -0.004 |
| Aridity | 0.115 | 0.057 | 0.003 | 0.227 |
| log(distance to protected areas) | 0.002 | 0.006 | -0.009 | 0.014 |
| log(nighttime lights) | 0.118 | 0.009 | 0.100 | 0.136 |
| Spatial range (3/$\phi$) | 3.823 | 0.564 | 2.893 | 5.096 |
| Spatial variance ($\sigma_{\omega}^{2}$) | 0.530 | 0.075 | 0.401 | 0.694 |
| iid variance ($\sigma_{\epsilon}^{2}$) | 0.370 | 0.010 | 0.350 | 0.390 |

**Net attendance rate for secondary school (boys)**

|  | **Mean** | **SD** | **2.5%** | **97.5%** |
| --- | --- | --- | --- | --- |
| (Intercept) | 1.384 | 0.154 | 1.088 | 1.691 |
| Slope | -0.005 | 0.003 | -0.011 | 0.001 |
| Distance to highways | -0.010 | 0.003 | -0.015 | -0.004 |
| Distance to water sources | -0.001 | 0.001 | -0.003 | 0.002 |
| Net primary production | -0.091 | 0.014 | -0.119 | -0.063 |
| Aridity | 0.120 | 0.051 | 0.019 | 0.220 |
| log(distance to protected areas) | -0.002 | 0.006 | -0.014 | 0.008 |
| log(nighttime lights) | 0.070 | 0.009 | 0.053 | 0.087 |
| Spatial range (3/$\phi$) | 4.021 | 0.301 | 3.485 | 4.665 |
| Spatial variance ($\sigma_{\omega}^{2}$) | 0.437 | 0.050 | 0.352 | 0.548 |
| iid variance ($\sigma_{\epsilon}^{2}$) | 0.340 | 0.010 | 0.322 | 0.361 |

**Child marriage (15 years old)**

|  | **Mean** | **SD** | **2.5%** | **97.5%** |
| --- | --- | --- | --- | --- |
| (Intercept) | -4.407 | 1.019 | -6.407 | -2.408 |
| Distance to highways | 0.004 | 0.002 | 0.000 | 0.008 |
| Distance to water sources | -0.001 | 0.001 | -0.003 | 0.001 |
| log(nighttime lights) | -0.060 | 0.005 | -0.069 | -0.051 |
| log(evapotranspiration) | 0.348 | 0.139 | 0.075 | 0.621 |
| Spatial range (3/$\phi$) | 3.168 | 0.447 | 2.365 | 4.117 |
| Spatial variance ($\sigma_{\omega}^{2}$) | 0.168 | 0.022 | 0.129 | 0.215 |
| iid variance ($\sigma_{\epsilon}^{2}$) | 0.045 | 0.004 | 0.038 | 0.052 |

**Child marriage (18 years old)**

|  | **Mean** | **SD** | **2.5%** | **97.5%** |
| --- | --- | --- | --- | --- |
| (Intercept) | -1.812 | 0.899 | -3.576 | -0.047 |
| Distance to highways | 0.007 | 0.002 | 0.003 | 0.010 |
| Net primary production | -0.067 | 0.009 | -0.084 | -0.050 |
| Aridity | -0.188 | 0.035 | -0.256 | -0.119 |
| log(nighttime lights) | -0.095 | 0.005 | -0.105 | -0.085 |
| log(evapotranspiration) | 0.194 | 0.121 | -0.044 | 0.431 |
| Spatial range (3/$\phi$) | 4.084 | 0.515 | 3.105 | 5.120 |
| Spatial variance ($\sigma_{\omega}^{2}$) | 0.309 | 0.045 | 0.230 | 0.405 |
| iid variance ($\sigma_{\epsilon}^{2}$) | 0.135 | 0.004 | 0.128 | 0.142 |

**Female labour force participation**

|  | **Mean** | **SD** | **2.5%** | **97.5%** |
| --- | --- | --- | --- | --- |
| (Intercept) | -1.116 | 0.379 | -1.867 | -0.377 |
| Slope | 0.008 | 0.004 | -0.001 | 0.016 |
| Net primary production | 0.005 | 0.027 | -0.048 | 0.058 |
| log(nighttime lights) | -0.222 | 0.015 | -0.252 | -0.192 |
| log(precipitation) | 0.002 | 0.08 | -0.155 | 0.159 |
| Spatial range (3/$\phi$) | 2.445 | 0.334 | 1.846 | 3.157 |
| Spatial variance ($\sigma_{\omega}^{2}$) | 0.538 | 0.061 | 0.428 | 0.667 |
| iid variance ($\sigma_{\epsilon}^{2}$) | 0.766 | 0.024 | 0.721 | 0.816 |

**Experience of physical violence**

|  | **Mean** | **SD** | **2.5%** | **97.5%** |
| --- | --- | --- | --- | --- |
| (Intercept) | -2.019 | 0.321 | -2.647 | -1.388 |
| Slope | 0.001 | 0.005 | -0.009 | 0.011 |
| Distance to highways | 0.009 | 0.005 | -0.001 | 0.018 |
| Distance to water sources | -0.004 | 0.002 | -0.008 | 0.000 |
| Temperature | 0.004 | 0.001 | 0.001 | 0.006 |
| Aridity | -0.175 | 0.074 | -0.319 | -0.029 |
| log(nighttime lights) | -0.097 | 0.011 | -0.118 | -0.075 |
| Spatial range (3/$\phi$) | 2.921 | 0.486 | 2.091 | 3.996 |
| Spatial variance ($\sigma_{\omega}^{2}$) | 0.472 | 0.061 | 0.364 | 0.603 |
| iid variance ($\sigma_{\epsilon}^{2}$) | 0.581 | 0.021 | 0.541 | 0.625 |

**Women decision-making on her own health**

|  | **Mean** | **SD** | **2.5%** | **97.5%** |
| --- | --- | --- | --- | --- |
| (Intercept) | 1.239 | 0.152 | 0.946 | 1.542 |
| Distance to highways | -0.012 | 0.004 | -0.019 | -0.004 |
| Aridity | 0.267 | 0.068 | 0.132 | 0.398 |
| log(distance to protected areas) | -0.002 | 0.011 | -0.023 | 0.019 |
| log(nighttime lights) | 0.042 | 0.011 | 0.021 | 0.064 |
| Spatial range (3/$\phi$) | 2.044 | 0.481 | 1.129 | 2.953 |
| Spatial variance ($\sigma_{\omega}^{2}$) | 0.303 | 0.045 | 0.219 | 0.394 |
| iid variance ($\sigma_{\epsilon}^{2}$) | 0.687 | 0.035 | 0.633 | 0.768 |

**Children receiving vitamin A supplements**

|  | **Mean** | **SD** | **2.5%** | **97.5%** |
| --- | --- | --- | --- | --- |
| (Intercept) | -0.034 | 0.264 | -0.553 | 0.484 |
| Distance to water sources | -0.002 | 0.002 | -0.005 | 0.001 |
| Net primary production | -0.011 | 0.018 | -0.047 | 0.025 |
| Temperature | 0.003 | 0.001 | 0.002 | 0.005 |
| log(distance to protected areas) | -0.008 | 0.009 | -0.026 | 0.010 |
| Spatial range (3/$\phi$) | 2.659 | 0.438 | 2.033 | 3.717 |
| Spatial variance ($\sigma_{\omega}^{2}$) | 0.559 | 0.059 | 0.454 | 0.683 |
| iid variance ($\sigma_{\epsilon}^{2}$) | 0.613 | 0.035 | 0.538 | 0.671 |

**Comprehensive knowledge of HIV**

|  | **Mean** | **SD** | **2.5%** | **97.5%** |
| --- | --- | --- | --- | --- |
| (Intercept) | 7.841 | 6.019 | -3.860 | 19.747 |
| Distance to highways | -0.007 | 0.011 | -0.029 | 0.015 |
| Net primary production | 0.145 | 0.063 | 0.022 | 0.268 |
| Aridity | 0.099 | 0.139 | -0.170 | 0.375 |
| log(nighttime lights) | 0.103 | 0.032 | 0.040 | 0.166 |
| log(evapotranspiration) | -1.326 | 0.827 | -2.964 | 0.284 |
| Spatial range (3/$\phi$) | 12.172 | 5.685 | 4.781 | 26.499 |
| Spatial variance ($\sigma_{\omega}^{2}$) | 0.358 | 0.175 | 0.116 | 0.789 |
| iid variance ($\sigma_{\epsilon}^{2}$) | 1.141 | 0.057 | 1.032 | 1.254 |

# SI.8. Summary of model validation metrics for each health and development indicator calculated at 5kmx5km high-resolution using INLA.

| **In-sample validation** | **Correlation** | **RMSE** | **MAE** | **Bias** |
| --- | --- | --- | --- | --- |
| Low birth weight | 0.296 | 0.177 | 0.140 | 0.184 |
| Use of Contraception | 0.574 | 0.123 | 0.099 | 0.984 |
| ANC number of visits (4+) | 0.707 | 0.247 | 0.201 | 1.009 |
| ANC Timing of visits | 0.578 | 0.246 | 0.200 | 0.484 |
| ANC Urine sample taken | 0.542 | 0.116 | 0.061 | 2.022 |
| ANC Blood sample taken | 0.580 | 0.113 | 0.057 | 1.928 |
| Children stunting | 0.416 | 0.211 | 0.169 | 1.522 |
| Children wasting | 0.367 | 0.184 | 0.141 | -1.435 |
| Net attendance rate for secondary school (girls) | 0.492 | 0.214 | 0.171 | 0.165 |
| Net attendance rate for secondary school (boys) | 0.435 | 0.205 | 0.164 | 0.037 |
| Child marriage 15 years | 0.491 | 0.088 | 0.068 | -0.831 |
| Child marriage 18 years | 0.674 | 0.140 | 0.112 | 0.102 |
| Labour force participation (women) | 0.544 | 0.215 | 0.173 | -0.986 |
| Experience of physical violence | 0.525 | 0.204 | 0.163 | -1.062 |
| Decision making on health (women) | 0.421 | 0.194 | 0.155 | 0.112 |
| Children receiving vitamin A supplements | 0.452 | 0.303 | 0.253 | 0.378 |
| HIV Knowledge | 0.362 | 0.194 | 0.151 | -0.716 |

| **Out-of-sample validation** | **Correlation** | **RMSE** | **MAE** | **Bias** |
| --- | --- | --- | --- | --- |
| Low birth weight | 0.262 | 0.178 | 0.141 | 0.240 |
| Use of Contraception | 0.551 | 0.126 | 0.101 | 1.067 |
| ANC number of visits (4+) | 0.691 | 0.252 | 0.205 | 1.100 |
| ANC Timing of visits | 0.551 | 0.2506 | 0.2044 | 0.5151 |
| ANC Urine sample taken | 0.519 | 0.117 | 0.062 | 2.044 |
| ANC Blood sample taken | 0.560 | 0.115 | 0.058 | 1.950 |
| Children stunting | 0.390 | 0.213 | 0.171 | 1.523 |
| Children wasting | 0.329 | 0.186 | 0.144 | -1.384 |
| Net attendance rate for secondary school (girls) | 0.465 | 0.218 | 0.174 | 0.191 |
| Net attendance rate for secondary school (boys) | 0.407 | 0.208 | 0.166 | 0.012 |
| Child marriage 15 years | 0.455 | 0.090 | 0.069 | -0.859 |
| Child marriage 18 years | 0.657 | 0.143 | 0.115 | 0.082 |
| Labour force participation (women) | 0.485 | 0.224 | 0.180 | -0.832 |
| Experience of physical violence | 0.468 | 0.211 | 0.169 | -0.974 |
| Decision making on health (women) | 0.342 | 0.200 | 0.160 | 0.116 |
| Children receiving vitamin A supplements | 0.412 | 0.309 | 0.259 | 0.372 |
| HIV Knowledge | 0.325 | 0.196 | 0.154 | -0.224 |
